# Prevalence, one-year-incidence and predictors of carcinoid heart disease

**DOI:** 10.1186/s12947-023-00316-6

**Published:** 2023-09-26

**Authors:** Isabel Mattig, Maximilian Richard Franke, Rene Pschowski, Anna Brand, Karl Stangl, Fabian Knebel, Henryk Dreger

**Affiliations:** 1 Deutsches Herzzentrum der Charité, Department of Cardiology, Angiology and Intensive Care Medicine, Campus Charité Mitte, Berlin, Germany; 2 DZHK (German Centre for Cardiovascular Research), Partner Site Berlin, Berlin, Germany; 3 Berlin Institute of Health at Charité – Universitätsmedizin Berlin, BIH Biomedical Innovation Academy, Berlin, Germany; 4 Charité – Universitätsmedizin Berlin, corporate member of Freie Universität Berlin and Humboldt-Universität zu Berlin, Charitéplatz 1, D – 10117 Berlin, Germany; 5 Helios Klinikum Emil Von Behring, Lungenklinik Heckeshorn, Berlin, Germany; 6 Sankt Gertrauden Krankenhaus, Gastroenterologie, Berlin, Germany; 7 Sana Klinikum Lichtenberg, Innere Medizin II: Schwerpunkt Kardiologie, Berlin, Germany; 8 Deutsches Herzzentrum der Charité, Department of Cardiology, Angiology and Intensive Care Medicine, Campus Virchow Klinikum, Berlin, Germany

**Keywords:** Carcinoid syndrome, Carcinoid tumour, 5-Hydroxyindoleacetic acid

## Abstract

**Background:**

Carcinoid heart disease (CHD) caused by neuroendocrine tumours (NET) is associated with an increased morbidity and mortality due to valvular dysfunction and right sided heart failure. The present study aimed to assess the prevalence and one-year-incidence of CHD in NET patients. Tumour characteristics, laboratory measurements, and echocardiographic findings were evaluated to identify predictors of CHD manifestation.

**Methods:**

The study was an investigator-initiated, monocentric, prospective trial. Patients with NET without previously diagnosed CHD were included and underwent comprehensive gastroenterological and oncological diagnostics. Echocardiographic examinations were performed at baseline and after one year.

**Results:**

Forty-seven NET patients were enrolled into the study, 64% of them showed clinical features of a carcinoid syndrome (CS). Three patients presented with CHD at baseline and three patients developed cardiac involvement during the follow-up period corresponding to a prevalence of 6% at baseline and an incidence of 6.8% within one year. Hydroxyindoleacetic acid (5-HIAA) was identified to predict the occurrence of CHD (OR, 1.004; 95% CI, 1.001–1.006 for increase of 5-HIAA), while chromogranin A (CgA), and Kiel antigen 67 (Ki 67%) had no predictive value. Six patients with CHD at twelve-month follow-up revealed a tendency for larger right heart diameters and increased values of myocardial performance index (MPEI) at baseline compared to NET patients.

**Conclusion:**

The prevalence at baseline and one-year-incidence of CHD was 6–7%. 5-HIAA was identified as the only marker which predict the development of CHD.

**Graphical Abstract:**

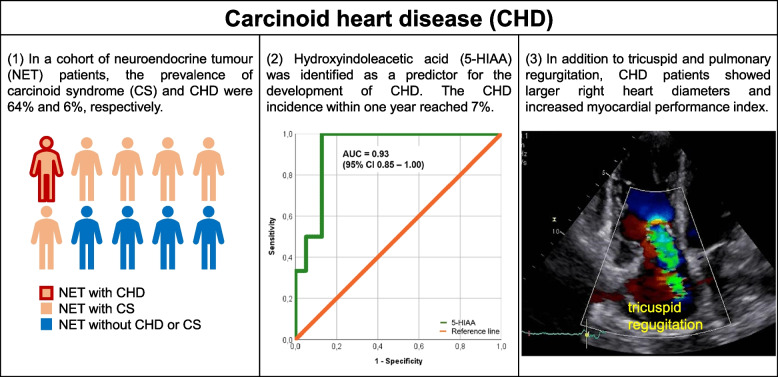

## Background

Carcinoid heart disease (CHD) caused by neuroendocrine tumours (NET) is associated with an increased morbidity and mortality due to right sided heart failure [1]. NETs occur with an incidence of 2.5 to 5 cases per 100.000 and are mostly localised in the gastrointestinal tract and less frequently in the lungs or ovaries [1, 2]. The release of vasoactive substances, like serotonin (5-hydroxytryptamine, 5-HT), prostaglandin, histamine and bradykinin, leads to carcinoid syndrome (CS), which is diagnosed in 30–40% of NET patients, and subsequently to CHD, which occurs in 50% of patients with CS [1, 3]. Three-year survival of patients with CHD is significantly reduced compared to NET patients without cardiac involvement (31% vs. 68%) [4]. Therefore, regular cardiological and echocardiographic monitoring is recommended by current guidelines [1].

Echocardiographic characteristics of CHD comprise regurgitation and stenosis of the tricuspid and pulmonary valve due to thickened and retracted leaflets [4]. Severe functional and structural remodelling of the tricuspid valve was found to be a major predictor of reduced survival [5]. Moreover, right atrium and ventricle are typically dilated and signs of volume and pressure overload of the right heart, such as a D-shaped left ventricle, may occur [4]. An involvement of the left heart (mostly in cases of septum defects with a right-to-left shunt), pericardial effusion, constrictive pericarditis or myocardial metastasis are less common [4, 6].

The incidence of CHD decreased due to treatment approaches like somatostatin analogues [1]. However, regression of cardiac involvement after development of CHD is rare [4, 7] and operative replacement of the affected tricuspid or pulmonary valve shows a thirty-day mortality of 9% and a median survival of 3 years [8]. Therefore, the evaluation and characterisation of predictors of CHD is an ongoing clinical need to identify high-risk NET patients.

The present study aimed to assess the prevalence and one-year-incidence of CHD in NET patients without previously diagnosed CHD. Moreover, we evaluated potential predictors of CHD manifestation, including tumour characteristics, laboratory tests and echocardiographic findings.

## Methods

### Study design

The study was an investigator-initiated, monocentric, prospective trial approved by the institutional ethics committee and performed in accordance with the Declaration of Helsinki. Each participant gave their written informed consent.

All patients underwent comprehensive gastroenterological and oncological diagnostics as recommended by the guidelines of the European Neuroendocrine Tumour Society (ENETS) [9, 10]. These diagnostics comprised laboratory measurements including chromogranin A (CgA) and hydroxyindoleacetic acid (5-HIAA), computed tomography (CT), magnetic resonance imaging (MRI), positron emission tomography CT scanning (PET) and/ or single photon emission CT (SPECT) as well as histopathological examination of the NET (Table 1).

**Table 1:** Baseline characteristics of the study cohort

	Overall cohort (*n* = 47)	Neuroendocrine tumours without carcinoid syndrome (*n* = 17)	Carcinoid syndrome only (*n* = 24)	Carcinoid heart disease at baseline (*n* = 3)	Carcinoid heart disease developed during follow-up (*n* = 3)
Male sex, n (%)	26 (55)	8 (47)	15 (63)	1 (33)	2 (67)
Age (IQR), years	61 (29–81)	56 (29–80)	64 (32–81)	65 (55–76)	63 (57–71)
**Localization of the tumour**					
Terminal ileum, n (%)	33 (70)	13 (76)	15 (63)	3 (100)	2 (67)
Small intestine, n (%)	2 (4)	0 (0)	2 (8)	0 (0)	0 (0)
Caecum/ appendix, n (%)	4 (9)	2 (12)	2 (8)	0 (0)	0 (0)
Cancer of unknown primary, n (%)	6 (13)	2 (12)	4 (17)	0 (0)	0 (0)
Lung, n (%)	1 (2)	0 (0)	0 (0)	0 (0)	1 (33)
Unknown, n (%)	1 (2)	0 (0)	1 (4)	0 (0)	0 (0)
**Metastasis**					
Liver, n (%)	35 (74)	11 (65)	18 (75)	3 (100)	3 (100)
Lymph nodes, n (%)	21 (45)	6 (35)	14 (58)	1 (33)	0 (0)
Bone, n (%)	9 (19)	4 (24)	2 (8)	2 (67)	1 (33)
Retroperitoneal, n (%)	3 (6)	0 (0)	3 (13)	0 (0)	0 (0)
Other, n (%)	15 (32)	8 (47)	4 (17)	1 (33)	2 (67)
None, n (%)	4 (9)	1 (6)	3 (13)	0 (0)	0 (0)
**Grading**					
G1, n (%)	22 (47)	8 (47)	12 (50)	1 (33)	1 (33)
G2, n (%)	22 (47)	8 (47)	10 (42)	2 (67)	2 (67)
G3, n (%)	0 (0)	0 (0)	0 (0)	0 (0)	0 (0)
Unknown, n (%)	3 (6)	1 (6)	2 (8)	0 (0)	0 (0)
**Ki67%**					
< 2%, n (%)	20 (43)	5 (29)	13 (54)	1 (33)	1 (33)
3–10%, n (%)	14 (30)	6 (35)	7 (29)	1 (33)	1 (33)
> 10%, n (%)	2 (4)	0 (0)	1 (4)	0 (0)	0 (0)
Unknown, n (%)	11 (23)	6 (35)	3 (13)	1 (33)	1 (33)
5-HIAA (IQR), µmol/24 h	106 (61–433) (*n* = 45)	65 (28–99) (*n* = 16)	189 (68–373) (*n* = 23)	Patient 1: 493; Patient 2: 2662; Patient 3: 558	Patient 4: 1836; Patient 5: 4945; Patient 6: 542
Cg A (IQR), ng/ml	198 (102–681) (*n* = 44)	86 (43–155) (*n* = 16)	243 (155–758) (*n* = 22)	Patient 1: 2129; Patient 2: 548; Patient 3: 4796	Patient 4: 1801; Patient 5: 3165; Patient 6: 6780
**Therapy**					
Surgical treatment, n (%)	29 (62)	15 (88)	13 (54)	0 (0)	1 (33)
Watch and wait, n (%)	2 (4)	1 (6)	1 (4)	0 (0)	0 (0)
Somatostatin analogues, n (%)	41 (87)	13 (76)	22 (91)	3 (100)	3 (100)
Chemotherapy, n (%)	1 (2)	1 (6)	0 (0)	0 (0)	0 (0)
Everolimus, n (%)	4 (9)	2 (12)	1(4)	0 (0)	1 (33)
Peptide receptor radionuclide therapy, n (%)	7 (15)	1 (6)	4 (17)	0 (0)	2 (67)
Others, n (%)	8 (17)	2 (12)	6 (25)	0 (0)	1 (33)

Continuous variables are shown as mean and standard deviation (SD) or median and interquartile ranges (IQR) depending on the skewness (uniform per variable)

*CgA* Chromogranin A, *G* Histological grade, *5-HIAA* Hydroxyindoleacetic acid, *Ki67%* Kiel antigen 67, *n* number

Echocardiographic measurements were performed in line with current guidelines of the American Society of Echocardiography (ASE), the European Association of Cardiovascular Imaging (EACVI) and the ENETS using a GE healthcare Vivid E9 (probe M5S, GE Healthcare, Vingmed, Horton, Norway) at baseline and one year after enrollment (range of follow-up period: nine to eighteen months) [11–14].

The assessment of right ventricular function and morphology comprised the parameters listed in Table 2. Right ventricular strain was measured according to the recommendations by Muraru et al. [15] using TOMTEC-Arena Version 2.41 (TOMTEC Imaging Systems GmbH, Unterschleissheim, Germany). To facilitate a more robust comparison, the analysis was restricted to patients for whom measurements were available at both baseline and follow-up assessments. Grading of valvular regurgitation was based on pressure half time, vena contracta and proximal isovelocity surface area method calculating the effective regurgitant orifice area and regurgitant volume. Valvular stenosis was quantified using transvalvular pressure gradients and peak velocities measured by continuous wave doppler. The tricuspid valve was evaluated in the apical four-chamber view and the right ventricular inflow-outflow view, the pulmonary valve in the parasternal short axis view. Carcinoid heart disease was diagnosed in case of typical echocardiographic findings as described by Davar et al. [1].

**Table 2:** Right heart function and morphology in NETs

	Baseline	Twelve-month follow-up	*P*-value
RVOT-VTI, cm (IQR)	15 (13–16)	14 (12–18)	0.937
TAPSE, mm ± SD	24 ± 4	25 ± 5	0.202
RV-Sʹ, cm/s (IQR)	13 (11–14)	13 (11–15)	0.500
MPEI (IQR)	0.45 (0.37–0.56)	0.42 (0.36–0.53)	0.959
Free wall RV strain, % ± SD	-27.3 ± 5.8, *n* = 30	-28.1 ± 7.2, *n* = 30	0.581
Global RV strain, % ± SD	-21.7 ± 4.9, *n* = 30	-22.5 ± 5.7, *n* = 30	0.478
RVD basal, mm ± SD	32 ± 7	34 ± 7	0.094
RVD mid, mm ± SD	25 ± 7	25 ± 7	0.872
RV apex-base, mm ± SD	73 ± 10	63 ± 11	0.020
RVOT1, mm ± SD	31 ± 4	30 ± 4	0.282
RVOT2, mm ± SD	23 ± 4	24 ± 4	0.392
RA area, cm^2^ (IQR)	13 (11–16)	14 (12–17)	0.112

Continuous variables are shown as mean and standard deviation (SD) or median and interquartile ranges (IQR) depending on the skewness (uniform per variable)

*MPEI* Myocardial performance index, *NET* Neuroendocrine tumour, *RA area* Right atrium area, *RV* Right ventricular, *RV apex-base* Right ventricle apex to base, *RVD* Right ventricular diameter, *RVOT* Right ventricular outflow tract, *RVOT-VTI* Right ventricular outflow tract velocity time integral, *RV-Sʹ* Systolic tricuspid annular velocity, *TAPSE* Tricuspid annular plane systolic excursion

### Statistical analysis

Statistical analysis was carried out in SPSS Statistics version 25 for Windows (IBM Corporation, New York, NY, USA) and Microsoft Excel version 16.41 (Microsoft, Redmond, Washington, USA). Categorical variables are listed as percentages. Continuous variables are presented as mean ± standard deviation (SD) or median with 25^th^ and 75^th^ percentile depending on their skewness (uniform per variable). Follow-up parameters were compared with baseline measurements in patients with NET (with or without concomitant CS) using the following tests: A t-test was performed to compare continuous variables with a normal distribution and a Wilcoxon signed rank test to compare not normally distributed continuous and categorical parameters. Patients with CHD were excluded from this analysis. A binary logistic regression analysis was used to identify predictors of CHD. For this purpose, patients with CHD at baseline and patients, who developed CHD in the follow-up period, were pooled in the CHD group and compared to patients with NET independently of concomitant CS. An additional receiver operating characteristic curve (ROC) analysis was used to assess the diagnostic value of 5-HIAA. A P-value of < 0.05 was defined as statistically significant. In addition, an exploratory analysis of patients with CHD was performed due to their small sample size.

## Results

Forty-seven patients with NET were enrolled into the study. The majority of patients (64%) showed clinical features of CS. At baseline, the prevalence of CHD was 6% in the entire NET group and 10% in NET patients with additional CS. Three additional patients developed cardiac involvement during the follow-up period corresponding to an incidence of 6.8% within one year. None of the patients died during the follow-up period. Baseline characteristics are summarized in Table 1 and echocardiographic measurements of the study cohort are listed in Tables 2, 3, and 4.

**Table 3:** Right heart parameters at baseline and twelve-month follow-up of patients with carcinoid heart disease at baseline

	Patient 1		Patient 2		Patient 3	
	Baseline	Follow-up	Baseline	Follow-up	Baseline	Follow-up
Duration from baseline to follow-up in months	10		11		11	
NYHA class	I	II	III	III	IV	II
NTproBNP, ng/l	395	245	940	1799	1128	2727
Therapy						
Cardiac intervention or operation (months since diagnosis)	None	Transcatheter pulmonary valve implantation (14)	None	Transcatheter pulmonary valve implantation (2)	None	Transcatheter pulmonary valve implantation (5)
Medication	ACE inhibitor, calcium antagonist, diuretics		ACE inhibitor, beta blocker, diuretics		ACE inhibitor, diuretics	
LVEF, %	72	69	60	60	53	53
RVOT-VTI, cm	31	18	14	10	N. A	39
TAPSE, mm	26	32	18	14	19	22
RV-Sʹ, cm/s	14	16	15	14	17	16
MPEI	1.40	0.73	1.38	0.72	0.31	0.84
Free wall RV strain, %	-28.2	-38.3	-31.3	-25.9	N. A	N. A
Global RV strain, %	-33.5	-33.0	-26.6	-25.6	N. A	N. A
RVD basal, mm	39	51	40	41	42	46
RVD mid, mm	27	43	34	37	36	41
RV apex-base, mm	82	79	61	56	65	61
RVOT1, mm	35	35	38	35	39	39
RVOT2, mm	29	30	26	29	22	24
RA area, cm2	28	32	25	23	21	19
TR	severe	severe	severe	severe	severe	severe
TS	none	none	none	moderate	none	none
PR	severe	none^a^	severe	mild^a^	moderate	severe^a^
PS	none	none^a^	N. A	none^a^	N. A	none^a^

*ACE inhibitor* Angiotensin-converting-enzyme inhibitor, *LVEF* Left ventricular ejection fraction, *MPEI* Myocardial performance index, *N. A*, Not available, *NTproBNP* N-terminal-pro hormone B-type natriuretic peptide, *NYHA* New York Heart Association, *PR* Pulmonary regurgitation, *PS* Pulmonary stenosis, *RA area* Right atrium area, *RV* Right ventricular, *RV apex-base* Right ventricle apex to base, *RVD* Right ventricular diameter, *RVOT* Right ventricular outflow tract, *RVOT-VTI* Right ventricular outflow tract velocity time integral, *RV-Sʹ* Systolic tricuspid annular velocity, *TAPSE* Tricuspid annular plane systolic excursion, *TR* Tricuspid regurgitation, *TS* Tricuspid stenosis

^a^Echocardiography was performed after patients had received transcatheter pulmonary valve implantation

**Table 4:** Right heart parameters at baseline and twelve-month follow-up of patients who developed carcinoid heart disease during follow-up

	Patient 4		Patient 5		Patient 6	
	Baseline	Follow-up	Baseline	Follow-up	Baseline	Follow-up
Duration from baseline to follow-up in months	14		12		12	
NYHA class	I	I	I	N. A	III	II
NTproBNP, ng/l	N.A	N.A	N.A	4053	544	643
Therapy						
Cardiac intervention or operation (months since diagnosis)	None	Surgical tricuspid valve replacement (63)	None	None	None	Transcatheter pulmonary valve implantation (38), surgical tricuspid valve replacement (41)
Medication	Angiotensin II receptor blocker, beta blocker, calcium antagonist, diuretics		Diuretics		ACE inhibitor, beta blocker, diuretics	
LVEF, %	55	51	62	60	53	59
RVOT-VTI, cm	N. A	10	38	11	12	N. A
TAPSE, mm	22	16	22	25	18	16
RV-Sʹ, cm/s	13	9	15	15	10	7
MPEI	0.63	0.51	0.32	0.76	0.55	0.34
Free wall RV strain, %	-26.2	-23.0	-31.6	-37.0	-17.1	-19.7
Global RV strain, %	-19.5	-19.5	-25.7	-30.7	-14.3	-17.8
RVD basal, mm	39	46	36	32	32	29
RVD mid, mm	40	38	29	27	32	25
RV apex-base, mm	96	68	68	82	69	73
RVOT1, mm	38	41	37	40	30	24
RVOT2, mm	29	32	17	35	23	21
RA area, cm2	16	32	14	19	20	18
TR	mild	None^a^	mild	moderate	moderate	mild^a^
TS	none	none^a^	none	none	none	none^a^
PR	none	moderate	none	none	moderate	severe^b^
PS	none	none	none	none	none	none^b^

*ACE inhibitor* Angiotensin-converting-enzyme inhibitor, *LVEF* Left ventricular ejection fraction, *MPEI* Myocardial performance index, *N. A*, Not available, *NTproBNP* N-terminal-pro hormone B-type natriuretic peptide, *NYHA* New York Heart Association, *PR* Pulmonary regurgitation, *PS* Pulmonary stenosis, *RA area* Right atrium area, *RV* Right ventricular, *RV apex-base* Right ventricle apex to base, *RVD* Right ventricular diameter, *RVOT* Right ventricular outflow tract, *RVOT-VTI* Right ventricular outflow tract velocity time integral, *RV-Sʹ* Systolic tricuspid annular velocity, *TAPSE* Tricuspid annular plane systolic excursion, *TR* Tricuspid regurgitation, *TS* Tricuspid stenosis

^a^Echocardiography was performed after patients had received tricuspid valve replacement and/ or

^b^transcatheter pulmonary valve implantation

### Echocardiographic characteristics of CHD patients

**Fig. 1:**
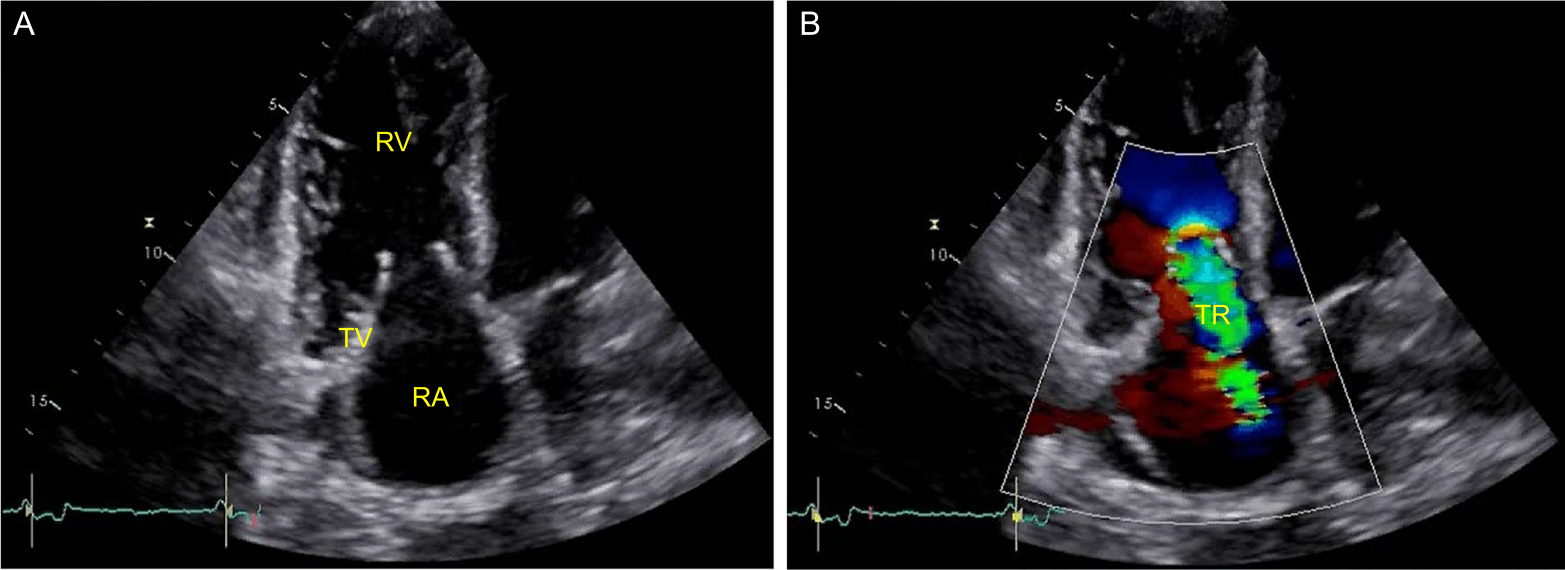
Echocardiographic imaging of the right heart with thickened, retracted, and immobile tricuspid valve leaflets (**A**) resulting in severe regurgitation (**B**) in a patient with carcinoid heart disease (apical four-chamber view), RA, right atrium; RV, right ventricle; TV, tricuspid valve; TR, tricuspid regurgitation

Patients with CHD at baseline suffered from moderate to severe tricuspid or pulmonary regurgitation at baseline and after twelve months (Fig. 1). One patient with CHD at baseline developed moderate tricuspid stenosis after twelve months, while the other five patients with CHD at twelve-month follow-up had no additional tricuspid or pulmonary stenosis. During the follow-up period, all three patients with CHD at baseline were treated with transcatheter pulmonary valve implantation resulting in an improved or stable New York Heart Association (NYHA) class (Table 3). Two patients, who developed CHD during follow-up, presented progressive valvular deterioration, which was treated by surgical tricuspid valve replacement and transcatheter pulmonary valve implantation. Following valve therapy, NYHA class improved within a short period of time (Table 4).

Comparison of right heart function and morphology of NET patients without CHD and all six patients, who had or developed cardiac involvement during follow-up, revealed a tendency for larger right atrial und ventricular diameters, lower values of longitudinal right ventricular function measured by the tricuspid annular plane systolic excursion (TAPSE), and elevated values of myocardial performance index (MPEI) as a marker of global systolic and diastolic right ventricular function in the CHD group at baseline. No further differences in echocardiographic measurements were observed.

### Predictors of CHD

**Fig. 2:**
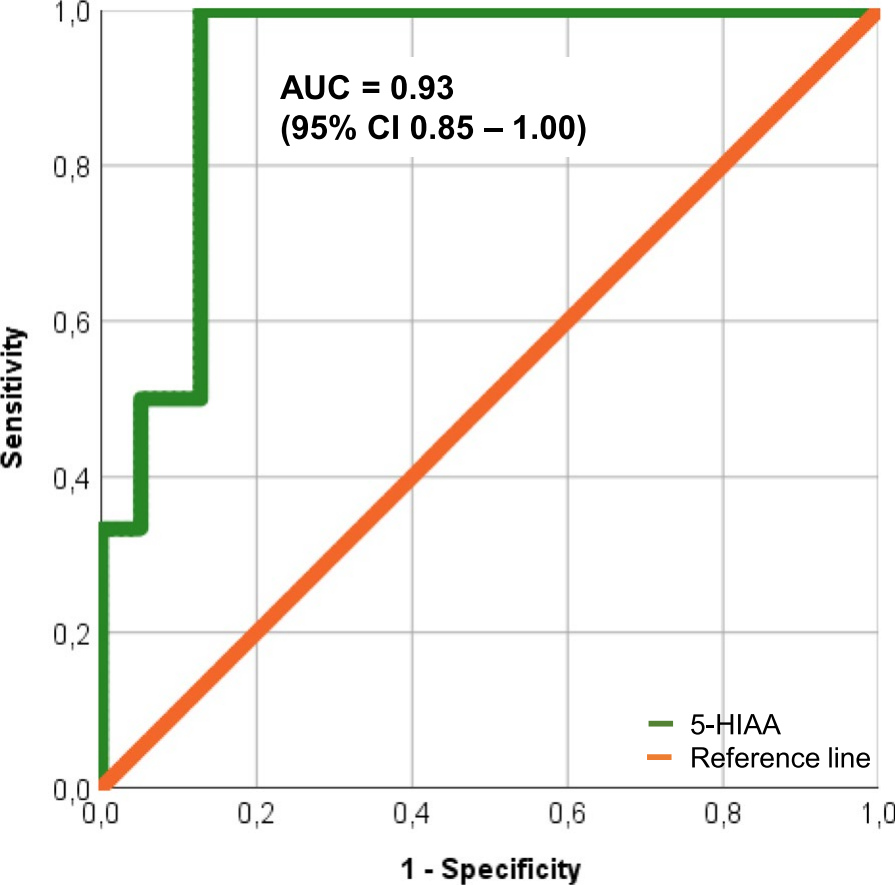
ROC analyses of hydroxyindoleacetic acid (5-HIAA) to discriminate patients with neuroendocrine tumours (NET) without carcinoid heart disease (CHD) and NET patients with CHD manifestation (green line, 5-HIAA; red line, reference line), AUC, area under the curve; CI, confidence interval

Six patients presented with CHD at twelve-month follow-up. Three of them had no signs of cardiac involvement at baseline. Mean 5-HIAA levels were identified to predict CHD manifestation (OR, 1.004; 95% CI, 1.001–1.006 for increase of 5-HIAA). In ROC analysis, the 5-HIAA cut-off value of 433 µmol/24 h corresponded to a sensitivity of 100% and specificity of 87% (Fig. 2). CgA and Kiel antigen 67 (Ki 67%) did not correlate with CHD at baseline and the occurrence of CHD within one year (Table 5).

**Table 5:** Predictors of CHD at baseline and the occurrence of CHD within one year

	*p* Value	Odds ratio (95% confidence interval)
5-HIAA	0.007	1.003 (1.001–1.005)
CgA	0.816	1.000 (1.000–1.000)
Ki67%	0.584	1.214 (0.606–2.434)

*CgA* Chromogranin A, *5-HIAA* Hydroxyindoleacetic acid, *Ki67%* Kiel antigen 67

## Discussion

In the present prospective study, the prevalence of CHD at baseline was 6% in NET and 10% in CS patients. The one-year-incidence reached 6.8% in NET patients. The occurrence of CHD was low compared to other studies, which detected a CHD prevalence ranging from 20 to 56% in CS patients [4, 16]. In contrast to our study participants, patients with CHD were treated less frequently with somatostatin analogues in these trials [4, 16]. Clement et al. hypothesized that somatostatin analogues lead to a lower prevalence of CHD due to serotonin reduction [10]. Therefore, the large number of treated patients may have had an influence on the detected incidence and prevalence of CHD in the present study. The mean duration from diagnosis to echocardiography was 49 months (± 54,9 months SD) in our study cohort − comparable to the aforementioned studies [4, 16].

In line with our echocardiographic measurements, patients with CHD in case series and reports mainly suffer from tricuspid regurgitation [4, 17, 18]. Echocardiographic examinations revealed thickened, retracted, and immobile valve leaflets resulting in an insufficient closure with regurgitation and in some cases additional stenosis [4, 17, 18]. Similar pathology is known in pulmonary regurgitation and stenosis, which is the second most commonly affected valve [4]. Patients who developed CHD during follow-up also had mild to moderate tricuspid regurgitation at baseline, possibly indicating the onset of CHD. Right ventricular and atrial dilatation were more frequent in patients with CHD at baseline compared to patients who developed CHD during follow-up. The more severe valve lesions in patients with CHD likely result in additional secondary remodelling of the right heart leading to further dilatation. Right ventricular enlargement was also detected in an observational study with 89 NET patients and 50 healthy controls by Haugaa et al. [19]. In addition to RV enlargement, the change in RV apex to base diameter at follow-up may also be explained by image foreshortening.

Elevated MPEI values in patients with CHD suggest a reduced global systolic and diastolic function. Haugaa et al. reported increased MPEI, decreased TAPSE and reduced right ventricular myocardial strain as typical characteristics of patients with CS in comparison to healthy controls [19]. A tendency to elevated MPEI levels was also observed in CHD patients compared to controls, but did not reach significance [19]. The authors assumed plaque fibrosis of the mural endocardium as the underlying cause of the impaired right heart function [19]. Additional myocardial fibrosis was not detected on magnetic resonance imaging in CHD [2, 17]. The results are consistent with our findings of increased MPEI and lower values of TAPSE in six patients with CHD in comparison to NET patients without cardiac involvement. As functional parameters are known to be load dependent, severe valvular regurgitation and stenosis play a further role in worsening right ventricular function [20]. Therefore, further deterioration of cardiac function in the course of the disease is likely and evaluation of different parameters of right ventricular function and morphology is recommended [21].

CHD patients of the present study were treated either surgically or interventionally based on their surgical risk (Tables 3 and 4). In high risk patients, our general approach was to treat the pulmonary valve interventionally and then re-assess the patient’s symptoms. If severe symptoms remain, patients were offered beating heart surgical tricuspid valve replacement via a lateral thoracotomy which – compared with both tricuspid and pulmonary valve replacement – substantially reduces perioperative morbidity and mortality. Interestingly, many patients report a substantial benefit of isolated interventional pulmonary valve replacement resulting in conservative management of tricuspid regurgitation. Patients with an interventional therapy of the pulmonary valve showed an improved valvular function at twelve-month follow-up with a positive impact on right heart remodelling and function in addition to an increase in physical capacity. However, most likely due to the persistent NET, two patients developed a progressive degeneration of the pulmonary bioprosthesis including a leaflet prolapse in one case resulting in severe regurgitation within a few months after implantation. Degeneration of the biological valve prostheses with a mechanism similar to that of native heart valves appears plausible in this context and thus, valve implantation may be futile in some patients. Regarding the tricuspid valve, CE-certified interventional therapies were not yet available. New interventional approaches, such as transfemoral transcatheter tricuspid valve replacement for severe tricuspid regurgitation, may provide new treatment possibilities for CHD patients who are unfit for surgery [22].

The pathophysiology of CHD is based on vasoactive substances released from NETs [1]. Serotonin binds on 5-hydroxytryptamine receptors (especially 5-HT_2B_) and induces proliferation of fibroblasts and myocytes as well as inflammation [1]. This results in the deposition of plaques on the endocardial tissue including valve leaflets, subvalvular apparatus, the atrium and ventricle [1]. Mean 5-HIAA predicted the occurrence of CHD in our study cohort (Fig. 2). Elevated 5-HIAA values are known to be associated with the development, but also with the progression, of CHD [23–26]. Consequently, the resection of hepatic metastases resulted in a stable cardiac disease [27]. All six CHD patients in our study cohort suffered from liver metastases; however, this did not predict CHD manifestation according to a previous study [24]. Current recommendations comprise echocardiographic evaluation of CHD every three to six months, depending on clinical presentation and laboratory measurements, including N-terminal-pro hormone B-type natriuretic peptide (NTproBNP) and 5-HIAA [1]. In our study cohort, patient four developed severe tricuspid regurgitation requiring surgical valve replacement within one year corresponding to increased 5-HIAA levels of 1836 µmol/24 h at baseline. Therefore, measurement of 5-HIAA should be considered to assess possible cardiac involvement and to initiate further cardiac evaluation.

Our results are limited by the small sample size of CHD patients. Therefore, an exploratory analysis was carried out. In addition, the time between diagnosis and echocardiography was variable, so subtle differences in functional capacity, cardiac morphology and function may not have been recorded.

## Conclusion

To conclude, the prevalence at baseline and incidence within one year of CHD was low. Only 5-HIAA predicted the occurrence of CHD. CHD was characterized by an impaired tricuspid and pulmonary valve function, resulting in valvular regurgitation and stenosis and right heart dilatation. Treatment with transcatheter pulmonary valve implantation and/ or tricuspid valve replacement led to improved or stable functional capacity. Therefore, regular echocardiographic follow-up is recommended in NET patients to detect valvular deterioration at an early stage and prevent right heart failure.

## Data Availability

The data that support the findings of this study are available from the corresponding author upon reasonable request.

## Abbreviations

5-HIAA: Hydroxyindoleacetic acid
5-HT: 5-Hydroxytryptamine
ASE: American Society of Echocardiography
CgA: Chromogranin A
CHD: Carcinoid heart disease
CS: Carcinoid syndrome
CT: Computed tomography
EACVI: European Association of Cardiovascular Imaging
ENETS: European Neuroendocrine Tumour Society
Ki 67%: Kiel antigen 67
MPEI: Myocardial performance index
MRI: Magnetic resonance imaging
NET: Neuroendocrine tumour
NYHA: New York Heart Association
PET: Positron emission tomography CT scanning
ROC: Receiver operating characteristic curve
SPECT: Single photon emission CT
TAPSE: Tricuspid annular plane systolic excursion
